# Confluence of Cellular Degradation Pathways During Interdigital Tissue Remodeling in Embryonic Tetrapods

**DOI:** 10.3389/fcell.2020.593761

**Published:** 2020-10-23

**Authors:** Juan A. Montero, Carlos I. Lorda-Diez, Juan M. Hurle

**Affiliations:** Departamento de Anatomiìa y Biologiìa Celular and Instituto de Investigación Sanitaria Valdecilla (IDIVAL), Universidad de Cantabria, Santander, Spain

**Keywords:** programmed cell death, apoptosis, extrinsic pathway, mitochondrial pathway, lysosomes, cell death genes, developmental senescence, tendon differentiation

## Abstract

Digits develop in the distal part of the embryonic limb primordium as radial prechondrogenic condensations separated by undifferentiated mesoderm. In a short time interval the interdigital mesoderm undergoes massive degeneration to determine the formation of free digits. This fascinating process has often been considered as an altruistic cell suicide that is evolutionarily-regulated in species with different degrees of digit webbing. Initial descriptions of interdigit remodeling considered lysosomes as the primary cause of the degenerative process. However, the functional significance of lysosomes lost interest among researcher and was displaced to a secondary role because the introduction of the term apoptosis. Accumulating evidence in recent decades has revealed that, far from being a unique method of embryonic cell death, apoptosis is only one among several redundant dying mechanisms accounting for the elimination of tissues during embryonic development. Developmental cell senescence has emerged in the last decade as a primary factor implicated in interdigit remodeling. Our review proposes that cell senescence is the biological process identified by vital staining in embryonic models and implicates lysosomes in programmed cell death. We review major structural changes associated with interdigit remodeling that may be driven by cell senescence. Furthermore, the identification of cell senescence lacking tissue degeneration, associated with the maturation of the digit tendons at the same stages of interdigital remodeling, allowed us to distinguish between two functionally distinct types of embryonic cell senescence, “constructive” and “destructive.”

## Introduction

Embryonic development, is a mostly plastic biological process that requires coordinate structural and architectural changes in cellular constituents. Among these changes are cell migration from one to other embryonic regions, differential proliferation of progenitors in specific embryonic regions, dissociation of epithelial tissues to form free mesenchymal progenitors, and aggregation and subsequent differentiation of cells to form organ primordia. It is believed that these cellular events are orchestrated at the local level by cell interactions mediated via secreted signals and/or changes in the composition and structure of the extracellular matrix that forms the substrate occupied by the cellular elements. Cell death is one additional embryonic process with a pivotal role in embryogenesis. As advanced by Glücksmann (1951) in the middle of the last century, cell death is associated with most embryonic events. However, in a high number of cases, cell death is massive and accounts for the elimination of a large mass of tissue that sculpt the shape of an embryonic organ (“morphogenetic cell death”), or of the whole embryonic primordium corresponding to an organ that is lost in the course of evolution (“phylogenetic cell death”). The formation of free digits in tetrapods or the loss of the tail in the larvae of amphibian Anura during metamorphosis are illustrative examples of processes characterized by massive cell death. The direct impact of massive cell death in morphogenesis and its precise and reproducible temporospatial pattern were often considered evidence of a singular developmental mechanism programmed at their genetic level. According to this view the prospective dying cells will die even if they are previously isolated from the embryo to explant cultures (Saunders, 1966). This belief was reinforced by the direct association of changes in the pattern of cell death with the diversification of organ morphology as species become evolutionarily adapted to serve different functional requirements. A most illustrative example of this fact are the varieties in the pattern of interdigital cell death according to the pattern of digit webbing in species adapted to live in distinct habits. Thus, among the differences in humans, mice or chickens, interdigital cell death is absent or reduced in the developing bat wings (Weatherbee et al., 2006), in the developing flippers of aquatic mammalias (Cooper et al., 2018) and in the feet of swimming aquatic birds (Tokita et al., 2020). The discovery of “cell death genes” directly related to the physiological elimination of specific cells in the worm *C. elegans* (Ellis and Horvitz, 1986) and their evolutionary conservation in mammals (Peter et al., 1997) provided strong support for this idea.

The idea of a singular genetic regulation of embryonic cell death was reinforced by the proposal of a specific type of cell death, termed “apoptosis,” (Kerr et al., 1972) accounting for cell elimination in most, if not all, physiological dying processes. However, research in the last decades have changed the view about the regulation of the embryonic cell death. Studies in a variety of models of embryonic tissue degeneration indicate that rather than being inherently programmed, cell death is locally regulated by extrinsic molecular interactions. The plasticity of the interdigital tissue in stages immediately prior to the onset of death provides solid support for the importance of extrinsic signals in the establishment of the degenerative process (see review by Montero et al., 2020). In addition, as will be discussed below, accumulating evidence has revealed that, far from being a unique way of embryonic cell death, apoptosis is only one among several redundant dying mechanisms accounting for the elimination of tissues in the course of embryonic development (Childs et al., 2014). Therefore, a detailed knowledge of the different dying cascades activated during each embryonic process would improve our understanding of the biological significance of cell death in developing systems.

## Areas of Interdigital Cell Death

**FIGURE 1:**
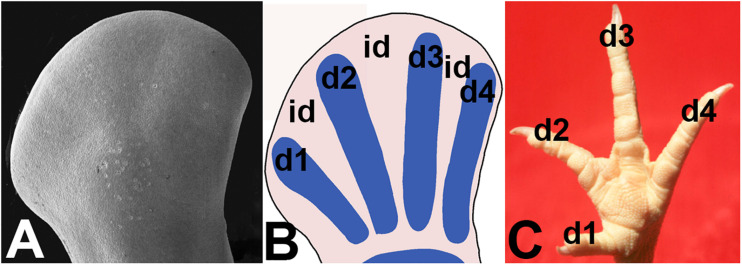
Illustrations showing the changes associated with digit formation in the avian autopod. **(A)** SEM micrograph of the autopod at the most initial stages of digit formation. **(B)** drawing showing the formation of digit rays (d1-d2-d3-d4) and interdigits (id) in the mesodermal core of the autopod. **(C)** Final morphology of the digits (d1-d2-d3-d4) in the adult chicken.

**FIGURE 2:**
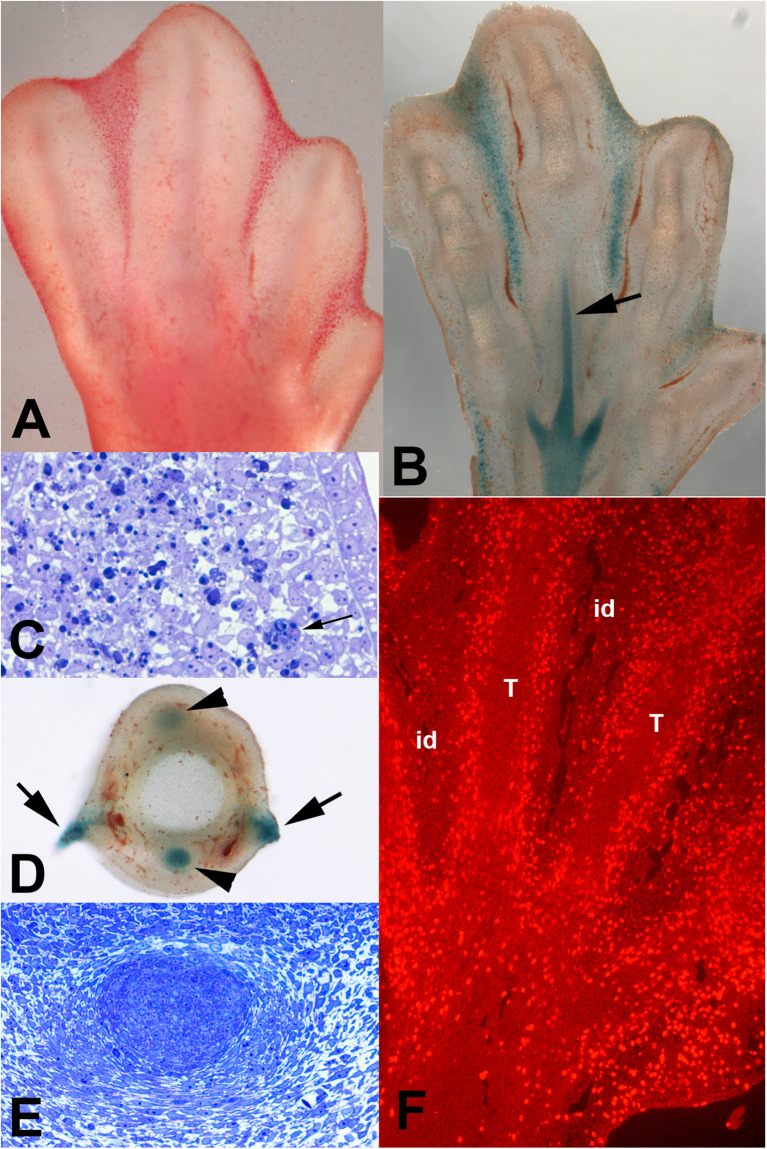
Patterns of cell death and cell senescence in the developing autopod of chick embryos at incubation day 7.5. **(A)** areas of cell death (INZs) mapped by vital stained with neutral red. **(B)** Vibratome section of autopod at stage equivalent to that illustrate in **(A)** showing the pattern of SAβ-gal staining for cell senescence. Note the identical pattern of SAβ-gal and neutral red vital staining in **(A)**. Note also the positivity for SAβ-gal in the developing tendons (arrow). **(C)** Semithin section of the third interdigital space stained with toluidine blue to show the structure of the regressing interdigits. Note the abundance of dark dying cells. Arrow shows a large macrophage containing phagocytosed dead cells. **(D)** Transverse vibratome section of digit 3 at id 8, showing SAβ-gal staining in the interdigital margins of the digit (arrows) and in the extensor and flexor tendons (arrow heads) in course of differentiation. **(E)** Transverse section of digit 3 stained with toluidine blue showing a detailed view of the digit flexor tendon. The level of the section is indicated by black arrows in **(B)**. **(F)** longitudinal section of the autopod through the level of the flexor tendons after incubation for 30 min in bromodeoxyuridine (BrdU). Note the reduced proliferation in the interdigital regions (id) and in the core of the developing tendons (T) (originally published in Lorda-Diez et al., 2009).

In developing vertebrates, digits are formed in the distal part of the limb primordium, termed the autopod, as radial prechondrogenic condensations (Figure 1). Between the differentiating digits rays, the mesodermal tissue remains undifferentiated, forming interdigital regions. The interdigital mesoderm undergoes degeneration once the digit primordia become established and progresses until the digits achieve their final morphology. This process lasts between 36 and 48 h in mouse and chick embryos and its sequence was traditionally mapped by vital staining with Neutral red or Nile blue (Figure 2A). Importantly, the intensity of the degenerating process in different species appears directly associated with the final morphology of the digits (Tokita et al., 2020). It is lower in species with webbed digits and very intense in species with free digits.

In this review we will survey the cell death effectors identified in the regressing interdigits of mammalian and avian species during the morphogenesis of the digits.

## Apoptosis

The term of “apoptosis” was coined to define, a type of cell death, “active, and inherently programmed,” that is distinct from “necrosis” (Kerr et al., 1972). In contrast to necrosis, cells undergoing apoptosis shrink and fragment without disintegrating their membranes and do not induce an inflammatory response (Kerr et al., 1972). The initial identification of regulatory genes, termed “cell death genes,” that activate or inhibited physiological cell death in the worm *C. elegans* (Ellis and Horvitz, 1986) and the subsequent identification of homologous genes in vertebrates, sparked an enormous interest in apoptosis in developmental and cancer studies (Peter et al., 1997). A family of cysteine-aspartic proteases termed caspases was identified as central players of apoptosis in vertebrates, although their functions were found to be much wider than those of apoptosis (McArthur and Kile, 2018). Caspases are produced as inactive zymogens that are sequentially activated by proteolysis through complex molecular cascades. Several *initiator* caspases, Caspase 8 and Caspase 9, have the function of activate the *executioner* Capases 3, 6, and 7. The executioner caspases cleave many cytosolic and nuclear substrates, resulting in, among other effects, the activation of the Caspase-activated DNase (CAD; Nagata et al., 2003) and irreversible death of the cell. A biochemical hallmark of the degradation process is the fragmentation of DNA in the internucleosomal regions (Wyllie, 1980). This feature was widely employed to identify the apoptotic process by electrophoresis and by the TUNEL assay.

**FIGURE 3:**
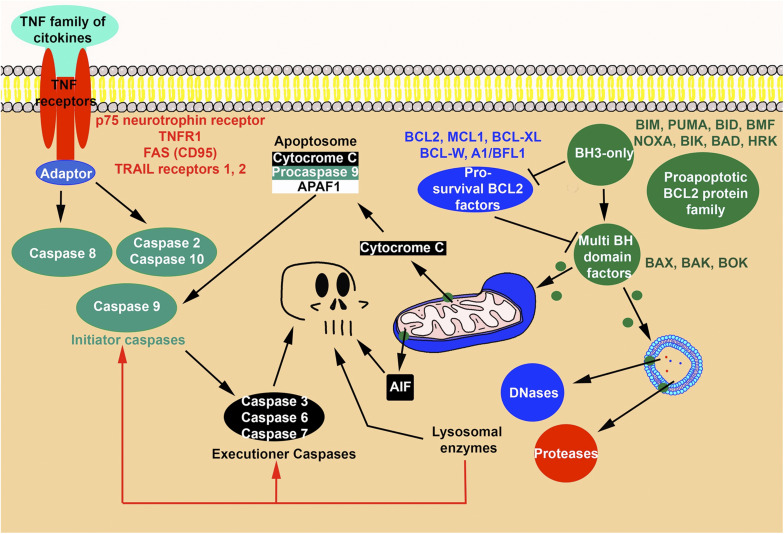
Degenerative routes during physiological cell death. The scheme illustrates the confluence of degenerative signals originated via the apoptotic mitochondrial pathway, the extrinsic apoptotic pathway and the lysosomal pathway. Note also, that mitochondria may deliver non-caspase apoptotic signals, such as AIF.

Two distinct apoptotic routes, extrinsic and intrinsic pathways, have been identified according to the signal that triggers the apoptotic molecular cascade (Figure 3). Regardless of the route, the apoptotic cells are TUNEL-positive and exhibit internucleosomal DNA fragmentation. These canonical features are observed at initial stages of interdigital tissue remodeling (Garcia-Martinez et al., 1993; Montero et al., 2016).

### Apoptosis Activated by the Intrinsic Pathway

The intrinsic route, also termed the *mitochondrial apoptotic pathway*, is characterized by permeabilization of the outer mitochondrial membrane. It is initiated following diverse stimuli affecting the cell or the cell environment. Many apoptotic-inducing stimuli recognized in different experimental settings to activate the intrinsic apoptotic pathway are associated with the remodeling of interdigits. These proapoptotic signals include the following: increased oxidative stress (Salas-Vidal et al., 1998; Eshkar-Oren et al., 2015), DNA damage (Montero et al., 2016), loss of cell-cell and/or cell-matrix adhesion (Zuzarte-Luis et al., 2006; Díaz-Mendoza et al., 2013), and growth factor withdrawal (Montero et al., 2001).

The initial stimuli activate a complex molecular machinery that involves the predominant participation of members of the BCL-2 protein family (gene homologous to *C. elegans* CED 9) characterized by sharing sequence and structural similarities. This gene/protein family comprises pro-apoptotic and pro-survival members (Figure 3). The two groups of factors have opposing effects that are translated to the outer mitochondrial membranes (Chen et al., 2015). The pro-apoptotic members can be grouped into two subgroups based on the number of BCL-2 homology domains (BH domains). Members of the first subgroup, termed BH3-only proteins, have only a BCL-2 homology domain (BH3), and include, BIM, PUMA, BID, BMF, NOXA, BIK, BAD, and HRK. The BH3-only factors become upregulated upon stimulation by the apoptotic signals mentioned above and block the pro-survival factors and/or activate directly the second subgroup of pro-apoptotic factors (multi-BH domain factors) constituted by BAX, BAK, and BOK (Ke et al., 2018) that cause permeabilization of the outer mitochondrial membrane and subsequent delivery of cytochrome C and mitochondrial genotoxic factors such as AIF (apoptotic inducing factor). Cytochrome C forms a complex with APAF-1 (apoptotic protease-activating factor-1) and pro-caspase 9, the “apoptosome,” which activates initiator Caspase-9. This caspase activates the executioner caspases.

The pro-survival factors of the BCL-2 family include BCL-2, MCL-1, BCL-XL, BCL-W, and A1/BFL-1. These factors block the activation of BAX, BAK, and BOK, by the BH3-only pro-apoptotic factors, thus protecting cells from apoptosis (Figure 3).

Preferential expression domains of pro-survival factors in the developing digits and pro-apoptotic factors in remodeling interdigits have been reported. Thus, BCL-2, BCLXL (Novack and Korsmeyer, 1994; Bečić et al., 2016), and A1 (Carrió et al., 1996) show preferential expression domains in the developing digits, while BAK and BAX are preferentially expressed in the interdigital regions (Dupé et al., 1999; Bečić et al., 2016).

Despite the importance of cell death in embryonic development, mice subjected to individual deletion of the different components of this cascade (including A1, Bak, Bax, Bad, Bcl2, Bik, Bim, Bmf, Bok, Hrk, Puma, and Noxa) lack the digit phenotype and most of them develop normally (Ke et al., 2018; and reviews by Tuzlak et al., 2016; Voss and Strasser, 2020). Syndactyly was only observed in double (Bax/Bak; Bax/Bim; Bim/Bmf) or triple (Bax/Bak/Bok) knockout mice (see Voss and Strasser, 2020). These observations indicate a major role of multi-BH domain factors in the regulation of this route of apoptosis in the interdigits. However, BAX and, most likely also BAK, are regulators of the lysosomal membrane permeability (Figure 3), suggesting that its deletion may not only interfere with the apoptotic pathway but also interfere with the lysosomal role in cell death (review by Johansson et al., 2010). The penetrance of syndactyly in double KO of Bak and Bax is accentuated in triple KO that includes the autophagic regulator gene Atg5 (Arakawa et al., 2017). This finding is consistent with a cooperative role of distinct death pathways in interdigit remodeling. This interpretation is also consistent with the absence of the syndactyly (Chautan et al., 1999) after deletion of APAF-1, a component of the apoptosome that exerts a pivotal function in the activation of Caspase 9. This initiator caspase is responsible for the activation of the executioner caspases and occupies the last step in the mitochondrial apoptotic pathway (Yoshida et al., 1998). Microscopic analysis showed that non-apoptotic cell death accounts for the removal of the interdigital cells in APAF-1 mutant mice (Chautan et al., 1999).

### Apoptosis Activated by the Extrinsic Pathway

Dying cells induced by this pathway are activated by signals external to the cell but are morphologically indistinguishable from apoptosis induced via the mitochondrial pathway (Figure 3). The receptors for the dying signal belong to a large superfamily of transmembrane receptors, termed the tumor necrosis receptor factor family (TNFR) comprising four major structurally homologous groups characterized by having an intracellular domain termed the death domain. The most representative members of these groups are p75^NTR^ neurotrophin receptor, TNFR1, FAS (CD95), and TRAIL Receptors 1 and 2 (Sessler et al., 2013). The ligands of this pathway are diverse, and most belong to the TNF family of cytokines. In the canonical pathway, upon ligand binding, the death domain recruits adaptor proteins, also containing a death domain sequence, forming a signaling complex with pro-caspase 8, resulting in its activation or repression. This initiator caspase, in turn, activates the executioner caspases (see review by Mandal et al., 2020). However, the complexity of this route is much greater. On the one hand, many members of the signaling cascade perform developmental functions other than promoting cell death (Sessler et al., 2013). On the other hand, two other initiator caspases, Caspase 10 and Caspase 2 may also participate in the signaling pathway and, most importantly, the extrinsic pathway may switch to other routes of cell death, including necroptosis and apoptosis via the intrinsic pathway (Mandal et al., 2020).

The implication of the extrinsic pathway in interdigital cell death has been proposed based on the specific expression of members of this cascade in the regressing interdigits. Thus, during the formation of the digits in mice, FAS and FASLG together with active Caspase 8 were preferentially expressed in the regressing interdigit (Svandova et al., 2017). In chick embryos, the immunoreactivity for the ligand TNF alpha was reported in the areas of mesodermal cell death of the developing limb (Wride et al., 1994) and nuclear immunoreactivity for active Caspase 2 was intense in interdigital apoptotic cells (Zuzarte-Luis et al., 2006).

In contrast to the expression patterns reported above, genetic approaches have failed to demonstrate the implication of this pathway in interdigital tissue remodeling. Mouse deficient in Caspase 8 are lethal before the formation of digits (Varfolomeev et al., 1998), and humans deficient in Caspase 10 lack the digit phenotype (Wang et al., 1999). Aditionally, despite of the expression of Caspase 2 in the interdigital cells, mice deficient in *Caspase 2* gene lack the digit phenotype (Bergeron et al., 1998), and interdigital electroporation of siRNA for Caspase 2 in chick embryos only delays, but does not inhibit, interdigit remodeling (Zuzarte-Luis et al., 2006). Together, these observations suggest that the above mentioned factors may be associated with other functions such as, remodeling of the extracellular matrix or as part of the engulfing and degradation of apopototic cells.

### Executioner Caspases

The executioner caspases 3, 6, and 7 are activated by both the intrinsic and extrinsic pathways and their proteolytic activity accounts for apoptotic cell degradation. Positivity for the active form of these three proteases in the interdigital dying cells along with the presence in the remodeling interdigits of their targets in the process of degradation supports the implication of apoptosis in interdigital tissue remodeling (Zuzarte-Luis et al., 2006). Although no syndactyly is observed in mouse subjected to caspase gene silencing (Kuida et al., 1996; Zheng et al., 1999), interdigital treatments with pan-caspase inhibitors provided evidence for a major implication of the executioner caspases in the physiological elimination of interdigital cells (Jacobson et al., 1996; Zuzarte-Luis et al., 2006). However, whether inhibition of cell death by these chemical treatments is partial or total remains to be fully clarified.

## Developmental Senescence and the Lysosomal Death Pathway: Same Process With Distinct Nomenclature?

The concept of cell senescence was originally formulated to describe the gradual loss of cell division capacity of cultured fibroblasts after several cell divisions (Hayflick, 1965). This finite mitotic capacity was explained as a protective mechanism secondary to the shortening of telomeres after several cell divisions (Harley et al., 1990) and was interpreted as a cell aging mechanism that avoid infinite proliferation of individual cells in multicellular organisms (replicative senescence). However, subsequent studies have shown that cell senescence is a common cell response that protects cells from physiological and/or pathological stresses including oncogene transformation (oncogene-induced senescence), hypoxia, oxidative stress, DNA damage or chemotherapeutic treatments. Consistent with this interpretation, senescence is a characteristic feature of benign and premalignant tumors and is induced in cancer tissues after cytotoxic treatments (Campisi and d’Adda di Fagagna, 2007). Cell senescence has been implicated in the pathogenesis of aging-related diseases (Baker et al., 2011) and is a common feature in degenerative diseases (Krizhanovsky et al., 2008; Muñoz-Espín and Serrano, 2014; Martínez-Cué and Rueda, 2020). The biological and biomedical interest in these observations promoted an enormous interest in the study and characterization of the cell senescence phenotype (reviewed by Gorgoulis et al., 2019). Cell cycle arrest is a major feature of cell senescence. This is associated with upregulation of tumor suppressor genes that block the progression of the cell cycle, such as p53, p16 (CDKN2A), and p21. Many morphological, structural, metabolic and molecular features are also common but are not fully specific characteristics of senescent cells. Two seminal markers additional to cell cycle arrest characterize senescence processes: (i) lysosomal hypertrophy; and (ii) activation of a secretory phenotype, termed SASP (senescence-associated-secretory-phenotype). The hypertrophy of lysosomes concerns the upregulation of most lysosomal enzymes (Kurz et al., 2000; Byun et al., 2009), but the detection of β-galactosidase at pH 6 (senescence-associated-β-galactosidase; SAβ-gal) is considered the most specific biomarker of cell senescence (Lee et al., 2006). The increased lysosomal activity is often associated with autophagy, which recycles cellular constituents providing energy and substrates required for the secretion of SASP components (Ivanov et al., 2013; see review by Shin and Zoncu, 2020). The SASP shows variations among senescent cells of different lineages, but common components of the SASP are proinflammatory cytokines and chemokines, growth factors, and matrix metalloproteinases. The SASP is thought to reinforce and spread senescence and activates immune responses that eliminate senescent cells (Acosta et al., 2013; Muñoz-Espín and Serrano, 2014).

In the last decade, in contrast to the protective role played by cell senescence in adult organisms, areas of massive cell senescence were observed during embryonic development in structures undergoing remodeling processes, such as the embryonic heart, otic vesicle, neural roof plate, limb primordia, or the mesonephros (Storer et al., 2013; Muñoz-Espín et al., 2013; Lorda-Diez et al., 2015, 2019; Varela-Nieto et al., 2019). It was proposed that cell senescence is primarily an embryonic process that is evolutionarily adapted to protect organisms from oncogenesis, premature aging and other cell stressors (Storer et al., 2013) via the elimination of unwanted or damaged cells (Muñoz-Espín and Serrano, 2014). In the embryonic models, the predominant view is that local remodeling signals promote cell senescence. However, the fate of senescent cells and their functional integration with other canonical embryonic degenerative processes, such as apoptosis, autophagy, extracellular matrix degeneration, or phagocytosis, await clarification (Childs et al., 2014; Li et al., 2018).

### Cell Senescence Is a Feature of the INZs

Interdigital tissue remodeling (formerly described as, interdigital necrotic zones, INZ) during digit morphogenesis constitutes the most appropriate model to study the biological significance of cell senescence in developing systems (Lorda-Diez et al., 2015). Consistent with the senescence nature of this morphogenetic process cell cycle arrest and overexpression of p21, p63, p73, and the Btg/Tob tumor suppressor gene family are precocious features of interdigit remodeling (Tone and Tanaka, 1997; Vasey et al., 2011; Lorda-Diez et al., 2015; Svandova et al., 2017). Furthermore, SAβ-gal histochemical staining is a very precise marker of INZs (Figure 2B), and also identifies all other areas of embryonic cell death (Muñoz-Espín et al., 2013; Storer et al., 2013; Lorda-Diez et al., 2019). Remarkably, the pattern of SAβ-gal is indistinguishable from the pattern of staining with vital dyes (Figures 2A,B) or with specific lysosomal markers, such as LysoTracker (Zucker and Rogers, 2019). Classical descriptions of the embryonic areas of cell death have relied mainly on vital staining procedures. The staining similarity between SAβ-gal and Neutral red or Nile blue is not surprising because vital dyes (Weijer et al., 1987), including LysoTracker (Zucker and Rogers, 2019), are markers of the lysosomal compartments (lysosomes, autophagosomes, or heterophagosomes).

Molecular compounds, such as tissue transglutaminase (tTG), which has been reported to be a marker of cell senescence (Kim et al., 2001), are specifically expressed in the developing interdigits (Dupé et al., 1999; Thomázy and Davies, 1999; Svandova et al., 2017). Importantly, immunohistochemistry and transcriptional analysis of INZ identified the prominent upregulation of characteristic SASP members (Dupé et al., 1999; Allan et al., 2000; Lorda-Diez et al., 2015; Svandova et al., 2017), including interleukin 8, Igf1, IgfBP5, HGF, Tgfβ2, AREGB (Amphiregulin B), matrix metalloproteinases (MMPs) (such as, MMP2, MMP9, MMP11, and ADAMTS9), and members of the TNF signaling pathway (such as TNF alpha, Fas (CD95), FasL, Tnfrsf1a, Tnfrsf21 (DR6), and Tnfrsf 23) (Table 1).

**TABLE 1:** SASP components identifiedin the regressing interdigits.

**Genes**	**6 id**	**7.5 id**	**8 id**
	**Expression fold changes**		
**Senescence-associated secretory phenotype (SASP; Lorda-Diez et al., 2015)**			
*IL8L1*	1.00 ± 0.04	11.10 ± 3.51*	20.59 ± 5.18*
*IL8L2*	1.02 ± 0.08	1.93 ± 0.22**	3.12 ± 0.46*
*AREGB*	1.05 ± 0.19	2.23 ± 0.43*	2.46 ± 0.71*
*HGF*	1.01 ± 0.08	1.98 ± 0.20**	1.85 ± 0.37*
*TGF*β*2*	1.03 ± 0.07	1.85 ± 0.25*	2.53 ± 0.53*
*IGF1*	1.00 ± 0.01	5.72 ± 1.62*	7.86 ± 2.20*
*IGFBP5*	1.00 ± 0.02	7.70 ± 1.86**	19.36 ± 4.44**
*MMP2*	1.01 ± 0.06	2.79 ± 0.45**	4.47 ± 0.78*
*MMP9*	1.00 ± 0.02	2.49 ± 0.44**	4.33 ± 0.91*
*Adamts9*	1.00 ± 0.04	6.70 ± 1.11**	22.66 ± 5.92*
*FAS*	1.02 ± 0.04	1.88 ± 0.18***	1.81 ± 0.24*
*TNFRSF21*	1.00 ± 0.02	0.83 ± 0.12	1.62 ± 0.25*
*TNFRSF23*	1.03 ± 0.14	1.92 ± 0.31*	3.28 ± 0.88*
**Other SASP components upregulated in the interdigit**			
*TNF*α	Wride et al., 1994		
*MMP11*	Dupé et al., 1999		
*tTG*	Dupé et al., 1999		
*FASLG*	Svandova et al., 2017		

The upper part of the table shows components undergoing intense transcriptional upregulation in the third interdigits of the chick limb. Note the increased expression from the stage preceding interdigit remodeling (id 6) to the stage of peak degeneration (id 8). The lower part of the table corresponds to characteristic SASP components reported to be expressed at high levels in the regressing interdigits of mouse and human embryos. ***p < 0.001; **p < 0.01; *p < 0.05 statistical significance (bold) in differences of gene expression levels between 7.5id (middle column) and 8id (right column) vs 6 id (left column).

### Cell Senescence and Tendon Formation During Digit Development

A central question about cell senescence in INZ (and also in other areas of embryonic cell death) is whether it is a degenerating event that precedes apoptosis or whether it is concurrent with apoptosis but plays a protective role against local stressors.

The pattern of SAβ-gal staining in vibratome sections of the developing autopod reveals two extremely different domains: the interdigits and the core of the differentiating tendon blastemas (Figures 2B,D). As described above, the interdigital regions are very positive for SAβ-gal. Notably, these domains have a characteristic doted appearance consistent with a distribution of SAβ-gal in lysosomes and phagosomes of most interdigital cells. By contrast, the labeling of the tendon blastemas is very intense but uniform, consistent with the presence of a dense number of primary lysosomes per cell. The senescence nature of the tendon domains is supported by cell cycle arrest in the core cells of the tendons (Figure 2F; Lorda-Diez et al., 2009) and by the precise expression of Tgfbeta 2 and CCN matricellular proteins (Lorda-Diez et al., 2013) and IGFBP-5 (Allan et al., 2000), which are characteristic SASP members that promote senescence in adult tissues and other embryonic models (Jun and Lau, 2010; Muñoz-Espín et al., 2013; Gibaja et al., 2019). Considering the different structures of the tendon blastemas and interdigits (Figures 2C,E) and the disparate fate of both tissues, it is likely that senescence in the tendon blastemas reflects a distinct biological function. Tendon blastemas in the stages of interdigital remodeling, are in the course of differentiation characterized by the production and maturation of a hypocellular fibrous matrix associated with the expression of CCN matricellular proteins (Lorda-Diez et al., 2011). It is tempting to propose the similarity of this process to that during cutaneous wound healing in adult organisms where senescence, regulated via CCN matricellular proteins, functions as a modulator of fibrosis (Jun and Lau, 2010). The occurrence of cell senescence associated with cell differentiation but not with apoptosis is not an exclusive feature of the developing tendons. During eye development, cell senescence identified by specific SAβ-gal labeling, p21 gene expression and proliferation arrest, has been reported in differentiating neurons and photoreceptors of the developing retina (de Mera-Rodríguez et al., 2019). Similar findings were observed in the maturing ventricular myocardium of embryonic mice (Lorda-Diez et al., 2019). Whether these non-degenerative processes positive for SAβ-gal constitutes “true” cell senescence processes or reflects that distinct biological phenomena share mechanistic events remains to be clarified. It is tempting to suggest a double and opposite functional significance of senescence in developing systems. In most cases, cell senescence is coincident with zones of cell death identifiable by current techniques of vital staining, that we propose to term “destructive developmental cell senescence,” to be distinguished from systems, such as the developing tendons or maturing retinal neurons, where senescence mechanisms appear associated with the establishment of low- or non-proliferative mature tissues. We propose the term “constructive developmental senescence” for these processes.

### Lysosomal Implication in Interdigit Remodeling and Cell Senescence

**FIGURE 4:**
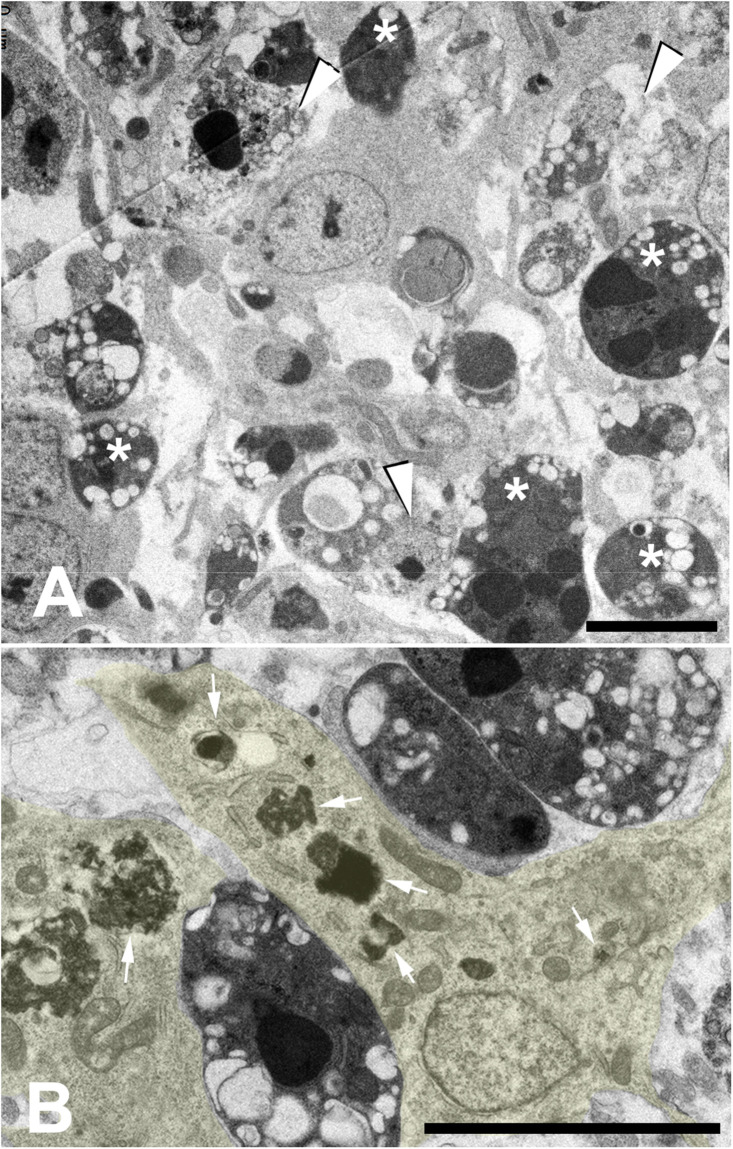
Transmission electron microscopic images of the interdigital tissue during remodeling (incubation day 7.5). **(A)** low magnification view showing the presence of electron-dense apoptosis (*) and necrotic like cells with disintegration of cell membranes (arrow heads). **(B)** healthy interdigital cells (enhanced in yellow) located between dark apoptotic cells. Arrows show the abundance a phagosomes of different sizes within the cytoplasm. Magnification bar = 5 μm.

The abundance of rounded dark dying cells in histological sections of interdigits during remodeling suggests that apoptosis is the predominant cell degenerative feature of this regressing tissue (Figure 2C). However, transmission electron microscopy showed that, in addition to dark apoptotic cells, necrotic-like cell remnants with disintegrated cell membranes (Figure 4A), and healthy cells (deduced by their nuclear morphology) rich in lysosomal vacuoles are very abundant (Figure 4B). These vacuoles are of variable size. In some cases, the small size, structure, and content of these vacuoles allow their identification as autophagic. In other cases, vacuoles are large and their content suggests a heterophagic origin. Cell senescence is accompanied by an increased mass of lysosomes (Kurz et al., 2000) and intense autophagy (Young et al., 2009). Additionally, at least in some cancer models of senescence, cells are highly enriched for genes related to phagocytosis and actively engulf neighboring senescent cells (Tonnessen-Murray et al., 2019). These observations together with the absence of other identifiable cell populations in the regressing interdigits, except for large macrophages of hemopoietic origin (Cuadros et al., 1992) and blood vessels, indicate that cells rich in autophagic and heterophagic vacuoles are the ones labeled with SAβ-gal; and therefore, they are senescent cells.

Studies devoted to analyzing the involvement of lysosomes in the areas of embryonic cell death provided compelling evidence for active lysosomal participation in remodeling limb tissues (Hurle and Hinchcliffe, 1978), albeit the knockdown of lysosomal genes in mice do not cause syndactyly (Saftig et al., 1995; Deussing et al., 1998; Roth et al., 2000). The following observations support the active participation of lysosomes in interdigit tissue removal: (1) as mentioned above, lysosomes and digestive vacuoles containing the cell remnants of auto and/or heterophagic origin are very abundant in the interdigital mesoderm, including TUNEL-positive apoptotic cells (Zuzarte-Luis et al., 2006); (2) various lysosomal cathepsins and lysosomal DNases are intensely upregulated in the interdigits at both the mRNA and protein levels, even when interdigits are explanted to culture dishes, to eliminate the participation of exogenous professional phagocytes of hematopoietic origin (Zuzarte-Luis et al., 2007; Montero et al., 2010); (3) the microenvironmental pH value in the interdigits decreases during remodeling to levels only appropriate for acidic enzymes (Montero et al., 2010); (4) lysosomal enzymes are released into the cytoplasm of cells during degeneration (Hurle and Hinchcliffe, 1978; Zuzarte-Luis et al., 2007; Montero et al., 2010); (5) local treatments with the cathepsin D inhibitor pepstatin A significantly increased the inhibition of interdigital cell death by treatments with pan-caspase inhibitors (Zuzarte-Luis et al., 2007); (6) syndactyly observed in Bak/Bax double-knockout mice, is potentiated when combined with the silencing of the autophagic regulator gene Atg5 (Arakawa et al., 2017).

Although autophagy and even heterophagy are survival mechanisms that supply energy to support the secretory profile of senescent cells (Young et al., 2009; Tonnessen-Murray et al., 2019), the above-reviewed observations suggest that rather than protecting cells from local stressors, cell senescence, via lysosomal activation exerts an active and potent catabolic function in the elimination of interdigital tissue.

### Lysosomal vs. Caspase-Dependent Dying Mechanisms: Sequential or Complementary Processes?

Whether the lysosomal- and caspase-dependent cell death are redundant and independent dying pathways, or if they constitute hierarchical mechanisms, are central questions to unravel the basis of interdigit remodeling. Studies of cell death during Drosophila metamorphosis provide examples of lysosomal cell death (autophagic) acting in parallel with caspases (salivary gland remodeling), independent of caspases (midgut cell death) and, upstream or downstream caspases in a context depend fashion (ovary maturation) (reviewed by Doherty and Baehrecke, 2018). In the case of the remodeling interdigits the identification of a common upstream regulation for both lysosomal cell death and apoptosis suggests a dual-parallel role of lysosomal and caspase-dependent cell death (Montero et al., 2016). DNA methyl transferases 1, 3A and 3B (DNMT1, 3A, and 3B) and the epigenetic regulators UHRF1 and 2 show high expression domains in the interdigital regions preceding the establishment of INZs. Gain- and loss-of-function experiments of DNMT3B and UHRF genes established a positive correlation between the expression level of these epigenetic regulators and cell death (Sanchez-Fernandez et al., 2019, 2020). Furthermore, interdigital progenitors are much more sensitive to genotoxic stimuli than cells of the growing digit tip that survive and account for digit outgrowth (Sanchez-Fernandez et al., 2020). Notably, local signals that are not harmful for differentiating progenitors cause DNA damage that interdigital cells try, but fail, to repair (Montero et al., 2016). According to these studies, failure to DNA repair triggers both apoptosis and lysosomal activation as a defensive response of cells against DNA damage. The sequence of lysosomal and caspase-dependent cell death that follows DNA damage after exposure to physiological (BMPs) or exogenous (H_2_O_2_) dying signals supports this interpretation (Montero et al., 2016). However, the complexity and promiscuity of the molecular machinery implicated in the different forms of cell death as well as the scarcity of interdigital phenotypes after gene silencing of the main players of each degenerative pathway, cannot rule out an alternative “hierarchical” function starting by caspase-dependent cell death and followed by lysosomal activation. Thus, mouse deficient in Apaf1, lacking caspase-dependent cell death, shows that apoptotic cells observed during physiological interdigit regression are substituted by cells with a rather necrotic appearance (Chautan et al., 1999). Whether this finding reflects that blocking caspases results in a context-dependent activation of lysosomes, as reported in fibroblast Bax/Bak^–/–^ (Shimizu et al., 2004), or if physiological lysosomal cell death expands and replace caspases in the elimination of interdigital cells has not been fully ascertain. However, as we reported above, triple KO of Atg5/Bax/Bak^–/–^ increases significantly the penetrance of syndactyly (i.e., the number of cells eliminated) observed in caspase-deficient mouse Bax/Bak^–/–^ (Arakawa et al., 2017), thus supporting the dual role of both dying pathways. Considering that lysosomal activation represents a manifestation of cell senescence it is tempting to suggest, that this double activation of caspases and lysosomes is the basis of what we called “destructive developmental cell senescence.”

## Additional Potential Functions of Cell Senescence in Interdigit Remodeling: Extrinsic Apoptotic Pathway, Recruitment of Macrophages and Extracellular Matrix Degradation

As described above, among the components of SASP identified in interdigit remodeling are members of the TNFR superfamily of transmembrane receptors, which participate in various models of apoptosis activated by the extrinsic pathway. Because full characterization of the interdigital SASP has not been performed to date, additional members of the TNF signaling pathway produced by the senescent interdigital cells may be involved in the regulation of cell death. Similarly, cytokines and chemokines included in the SASP may contribute to recruit professional macrophages (Cuadros et al., 1992) to fully accomplish the elimination of dying cells and cell detritus. This is an important, but dispensable, aspect of the regression of the interdigital tissue (Wood et al., 2000).

A final important feature of interdigit remodeling is the degradation of the extracellular matrix, collapse of the blood vessels (Hurle et al., 1985) and the subsequent elimination of waste ectodermal tissue into the amniotic sac (Hurle and Fernandez-Teran, 1983; McCulloch et al., 2009; Kashgari et al., 2020). This aspect of tissue remodeling has been often neglected in developmental studies devoted to cell death-mediated morphogenesis. However, its dysregulation abrogates the formation of free digits (McCulloch et al., 2009; Kashgari et al., 2020). The abundance of secreted matrix metalloproteases in the SASP (described above) sustains without doubt this important aspect of interdigital tissue remodeling.

## Concluding Remarks

The experimental data surveyed here support the occurrence of a unified process of embryonic tissue remodeling involving the activation of two main degenerative routes, namely, the lysosomal pathway-mediated and caspase-dependent cell death. From an historical point of view, the introduction of the term *apoptosis*, to define a type of cell death “active, and inherently programmed” distinct from “*necrosis*” (Kerr et al., 1972) led to a breakthrough in our knowledge of the control and biological significance of cell death in embryonic and non-embryonic processes. However, as a counterpart, the role of lysosomes was neglected, despite initial descriptions considering lysosomes as the primary cause of the embryonic remodeling processes (Salzgeber and Weber, 1966; Hurle and Hinchcliffe, 1978). The description of “*developmental senescence*” recovered lysosomes as major effectors of embryonic cell death, and the discovery of the SASP shed much light on our understanding of local signaling emanating from the degenerating cells that modulates the intensity and expansion of tissue remodeling. However, it is important to highlight the differences observed between two adjacent senescent processes occurring at the same stages in the developing autopod: interdigit remodeling and tendon tissue maturation. In contrast to reports in adult tissues where senescence is considered a protective process against stressors, interdigital senescence appears to be a destructive (Muñoz-Espín et al., 2013), but finely regulated process (“*destructive developmental cell senescence*”). By contrast, senescence in the developing tendons, is not associated with tissue degeneration and appears to be a constructive process that regulates tendon maturation (“*constructive developmental senescence*”).

## Author Contributions

JM, CL-D, and JH discussed, reviewed, edited, and wrote the manuscript. All authors contributed to the article and approved the submitted version.

## Conflict of Interest

The authors declare that the research was conducted in the absence of any commercial or financial relationships that could be construed as a potential conflict of interest.
